# Development, Validity, and Reliability of the Women’s Capabilities Index

**DOI:** 10.1080/19452829.2017.1422704

**Published:** 2018-01-25

**Authors:** Giulia Greco, Jolene Skordis-Worrall, Anne Mills

**Affiliations:** * Department for global Health and Development, London School of Hygiene and Tropical Medicine, London, UK; ** The Centre for Global Health Economics, Institute for Global Health, University College London, London, UK

**Keywords:** Capability approach, Gender, Health, Measurement, Well-being, Validity, Reliability, Outcome measure, Low- and middle-income countries, Developing countries

## Abstract

We report the results of a series of validity and reliability tests performed during the development of the Women’s Capabilities Index (WCI) in Malawi. The WCI is a multidimensional measure based on Sen’s capability framework for assessing women’s quality of life. Construct validity was assessed by investigating the expected relationships of the dimensions with key socioeconomic characteristics. The majority of hypothesized associations were found to be statistically significant in the expected direction. This provides evidence that the index is measuring quality of life as intended in the conceptual model. Further evidence in support of the index’s validity was given by the high degree of correlation between the WCI and another scale measuring comparable (but not identical) domains of quality of life. The results from the internal consistency and the test–retest repeatability also offered encouraging evidence on the reliability of the instrument. This is the first study to rigorously and comprehensively test for validity and reliability a capabilities index for a low-income setting. The results of the validity and reliability tests provide supportive evidence that a locally developed measure of capabilities can be used as a robust tool for the assessment of women’s quality of life.

## Introduction

Increasing attention has been given to the evaluation of complex interventions in public health. While the methods for the economic evaluation of clinical interventions are well established (Drummond 1997), the economic evaluations of public health interventions are scarce and raise additional methodological challenges (Weatherly 2009; Greco 2016). A common outcome indicator for the evaluation of public health programmes is the quality-adjusted life-years measure (QALY). The “quality of life” component is derived using preference elicitation techniques, such as the time trade-off (trading time in a particular health state with death) or standard gamble (chancing death and a health state). In low- and middle-income countries, a more widely used indicator is the disability-adjusted life-years measure (DALY). The disability component reflects how affected a person’s health would be with respect to a given health impairment.

It has been argued that DALYs or QALYs are not well suited to the assessment of public health and community-based initiatives; because they only focus on the health-related part of well-being, and thus they fail to capture adequately the full range and breadth of outcomes generated by these complex interventions Weatherly (2009).

With the use of the QALY or DALY, the benefits of many intersectoral interventions are therefore at risk of being underestimated. There is an expanding field of research that is advocating for the development of outcome measures based on a broader evaluative space. Sen’s capability approach offers a potential framework within which to build a multidimensional outcome measure (Coast et al. 2008; Lorgelly et al. 2010; Smith 2012; Simon 2013; Coast, Kinghorn, and Mitchell 2015; Greco 2016)

Sen’s main argument is that what matters in the evaluation of a good life are people’s capabilities: the abilities to achieve those things that people have reason to value (1993). These valuable “beings and doings” can range from basic functionings, such as being well nourished and living in a decent house, to more complex functionings such as being in control over personal decisions. The capability approach distinguishes itself from other conventional approaches such as utility, income or basic needs, because of its broader and more open evaluative space (Coast, Smith, and Lorgelly 2008).

Despite great interest in Sen’s work, a very limited number of capability measures have been developed and validated for practical use in the evaluation of public health policies (Mitchell et al. 2017). One of these is the ICECAP (ICEpop CAPability) set of measures of health and social care for the general adult population, for the older population and for end-of-life care in the UK and other high-income countries (Coast et al. 2008; Al-Janabi, Flynn, and Coast 2012).

The work reported here formed part of the first study that developed a capabilities metrics in a low-income setting, the Women’s Capabilities Index (WCI). The WCI is a multidimensional index based on Sen’s capability framework. It has been developed for assessing the quality of life of women in rural Malawi (Greco et al. 2015).

In order to be able to put more confidence in the results generated from its use, the WCI was submitted to a series of rigorous and comprehensive tests for content, construct, convergent and discriminant validity, and reliability. Evidence of these tests is reported in this paper.

## Background

### The WCI

The MaiMwana Project is a community-based participatory intervention that organizes groups of women in rural villages in Mchinji District, Malawi. Women meet to discuss, develop and implement strategies to overcome maternal and neonatal health issues. It has been demonstrated that the MaiMwana Project has reduced maternal mortality by 74% (Lewycka et al. 2010). However, some programme effects are likely to go beyond health and might have an impact on several aspects of women’s quality of life. The complexity of the likely outcomes of the MaiMwana Women’s Groups raised methodological challenges on the choice of outcome measure to use for its evaluation, hence the development of a multidimensional index.

The WCI was developed following several steps, as described in Greco (2016): (1) theoretical model: selection of capabilities; (2) measurement model: selection of capability indicators and questionnaire design; (3) building of the capability set for a sample of women through a survey; (4) aggregation of the indicators into one index; and (5) validation of the index.

The selection of capabilities was done adopting a participatory approach. The views, values, and opinions of 129 women from Mchinji, Malawi were collected through a series of focus group discussions (FGDs). Each “being and doing” that was valued by the participants as important in their lives was considered part of the capability list.

The selection of indicators and questionnaire design was done with the research and field team. A protocol describing in lay terms the theoretical foundation of the measure was presented and discussed with the team, with a proposition of a measurement model based on the re-elaboration of the lists of capabilities that were identified during the FDGs (Greco et al. 2015). The final index comprises 6 main capabilities (*physical strength, inner well-being, household well-being, community relations, economic security,* and *happiness*), with a set of sub-dimensions for a total of 26 sub-dimensions. The sub-dimensions were assessed with different indicators for a total of 72 variables as described in Table 1.

**Table 1.** WCI in Malawi

*Physical strength*: 4 sub-dimensions, 9 variables
being able to do physical work: physical health, energy
having enough food: types of food eaten in the last week
being able to avoid diseases: hygiene, HIV awareness, HIV protection, bed net use
being able to space births: family planning availability, FP practice
*Inner well-being*: 5 sub-dimensions, 11 variables
peace of mind: mental health, sleep lost, relax time
control over personal matters: control over daily activities, permission to go to funeral, permission to go to clinic
free from oppression: freedom of expression, lack of oppression
living without shame
knowledge: read, write
*Household well-being*: 5 sub-dimensions, 13 variables
free from domestic violence: domestic violence past, domestic violence likely in future
control over money: access household money, control over minor expenditure, control over major expenditure
living in a decent house: toilet, water, house tenure, fear of house eviction, house adequate, house adequate in 6 months
children’s education: all children will reach desired level of education
family care: take care of children and husband
*Community relations*: 5 sub-dimensions, 21 variables
access services: easy/difficult to reach health centre, under 5 clinic, school, market, water source, church
feeling safe and comfortable in the village: fear of witchcraft, moving away from village, safety village, assault past, assault future, theft past, theft future
being able to join community groups: groups available, group membership, position
social exclusion and discrimination: not allowed in groups, gender discrimination, poverty discrimination
being respected: respect, admiration
*Economic security*: 5 sub-dimensions, 16 variables
safety net: help asked to you, you asked for help
land: land ownership, fear of eviction
assets: bike, oxcart, ox, chicken, pig, goat, cow, radio, mobile, bed net
business opportunities: access to business opportunities
copying with shocks: able to feed the family if crisis
*Happiness*: 2 sub-dimensions, 2 variables
satisfaction: satisfied with life overall
happiness: taking all things together, how happy are you?

Four aggregation techniques were used to test the implications for the results of adopting different approaches. The dimensions and sub-dimensions were aggregated: (1) giving equal weight to each dimension and sub-dimensions, (2) assigning weights based on collective value judgments, (3) assigning weights derived from survey-based individual preferences, and (4) using statistical technique (principal component analysis).

March 2010 and took place in Mchinji district, Malawi. The capability set was built with a household survey for a sample of 258 mothers who had delivered their babies during the previous year. Details of the sampling strategy were described in Greco (2016). Informed consent was obtained from all individual participants included in the study.

### Validity and Reliability

Validity and reliability assessments are a critical step in the development of a measure. An instrument should be tested and found to be adequate for the research purposes: validity—in terms of whether the indicator is actually measuring what is supposed to measure; and reliability—in terms of estimating the degree of error inherent in the measurement (Atkinson et al. 2002; Lohr 2002; World Bank 2004; Ibrahim and Alkire 2007; Reeve et al. 2013).

There are two important aspects to be considered before setting up a validation process: the nature of what is being measured and the relationship of the observation to its intended cause (Kane 2006).

Since the particular nature of the variable “quality of life” does not allow for direct measurement like other variables such as blood pressure or income, no single instrument gives the right answer; hence a validity test is needed for assessing if the selected instrument can enable the researcher to make accurate inferences about an individual (Streiner and Norman 2008); or if the instrument is “fit for purpose.”

The aim of the validity tests is to address the question: to what extent is the instrument really measuring quality of life for women in rural Malawi?

There is no direct way to answer this; however, several types of validity assessments can contribute to it. The “Trinitarian” point of view advocates for the three Cs: content validity, criterion validity, and construct validity (Landy 1986).

Content validity (the extent to which the measure includes the most relevant and important aspects of a concept) is a critical phase in item construction for ensuring that the meaning attributed to each question is conveyed in the right manner. It is rated by ISOQOL^1^ members as one of the most important form of validity tests for the development of patient-reported outcome measures (Reeve et al. 2013).

Construct validity is the degree to which scores on the measure relate to other measures (e.g., patient-reported or clinical indicators) in a manner that is consistent with theoretically derived a priori hypotheses. It has been widely used to test health measurement scales and psychometric instruments, including quality of life indices (Bonomi et al. 2000; Webster 2010; Colbourn, Masache, and Skordis-Worrall 2012). In the capability literature, it has been used to validate the ICECAP measures (Al-Janabi et al. 2013).

Discriminant validity is the degree to which two measures that theoretically should not be related are in fact not related, while convergent validity refers to the degree to which two measures that theoretically should be related are in fact related.

In addition to this, an instrument should also be tested for reliability in order to estimate the degree of error that is intrinsic in any measure.

## Methods

### Content Validity

*Content validity* of the capability survey instrument was assessed during the pilot stage of the FGDs, through cognitive debriefing interviews with 20 women of childbearing age from the study site. These one-to-one interviews helped to determine whether concepts and items were understood by respondents in the same way that instrument developers intended. The cognitive debriefing interviews were conducted using two qualitative methods:

- think-aloud—respondents were asked to think aloud when answering the questions, in order to outline the process that generates the final response;
- paraphrasing—respondents were asked to repeat the questions in their own words.

These methods have been used extensively for the development of outcome measures (Bowden et al. 2002) and are a key component of the cognitive debriefing process. A description of the intended referential and connotative meaning for each of the survey questions was drawn up together with the field team. This process, in addition to guiding the assessment of content validity, also clarified the concepts for translation and use by other researchers, in the same or different contexts (Bowden et al. 2002).

Respondents were told that it did not matter what her responses to the questionnaire were, but rather that the researchers were interested in the mental process of understanding the question, and formulating the answers. The interviewers compiled detailed daily field reports which were compared and shared with the research team. Any change in the items or structure of the questionnaire was discussed within the team until agreement was reached.

After content validity was tested, the final version of the capabilities tool was administered with a survey in order to collect data on women’s capabilities. The WCI was then constructed for each woman in the sample, and it was subjected to a range of *validity* and *reliability* tests.

### Construct Validity

Following Cronbach and Meehl’s seminal work on validity in psychological tests (1955), the latent variable in this research (quality of life and its dimensions) was linked to relevant contextual variables by a hypothesis or construct, before performing the test. The hypothetical constructs were tested to see whether the instrument, compared to other measures, was performing as expected a priori.

Following Coast and Al-Janabi (Coast et al. 2008; Al-Janabi et al. 2013), the association between measured capabilities and background factors was investigated using chi-squared tests for ordered categorical variables, and compared with the hypothesized relationship. Where the number of cell counts was less than 5, Fisher’s exact tests were used when computationally feasible. Where it was not possible, values were grouped and variables were re-coded to increase cell counts.

Those background variables that were part of a dimension were not taken into consideration in the validity test of that particular dimension, for example, health is one component of the physical strength dimension; thus it was excluded from the association test. Alongside the direction of the association, the statistical strength of the evidence for each relationship was checked and reported using significance levels of 5% and 1%. All analyses were undertaken using Stata version 12.

The hypothesized constructs drew mainly from the extensive qualitative work undertaken during the development of the theoretical model and the selection of the capabilities (Greco et al. 2015). The development of the theoretical model was an important step in the construction of the WCI because it allowed women not only to explore in great depth people’s understanding of quality of life, but also to identify and value the different dimensions of quality of life and the factors that have an influence on it (Greco et al. 2015). Hence, the data collected provided a rich and solid base for building the hypothesized relationships. The constructs are detailed below and are summarized in Table 2.

**Table 2.** Summary of hypothesized associations between dimensions and conversion factors

Conversion factors	Type of variable	Physical strength	Inner well-being	Household well-being	Community relations	Economic security	Happiness
Rural area	Binary	None	None	None	None	Negative	None
Health	Ordered	n/a	Strong Pos	Positive	None	Strong Pos	Strong Pos
Education	Ordered	None	Strong Pos	None	Positive	Strong Pos	None
Age	Ordered	Negative	None	None	Positive	None	None
Married	Binary	Strong Pos	Ambiguous	Positive	Positive	Strong Pos	Positive
Wealth index	Ordered	Strong Pos	Strong Pos	Strong Pos	Positive	n/a	Positive

Note: n/a (not applicable): If the variable is part of the indicator, no association is estimated. Pos: positive.

#### Description of constructs

Women who live in rural villages compared to those living in urban or peri-urban areas are expected to face harder economic conditions due to the remoteness of the area and a lack of economic opportunities.

Women who are in good health are likely to score higher in most of the dimensions capturing quality of life since having an able body is a pre-condition for achieving the majority of the capabilities (Sen 2002). Positive associations would be expected with inner well-being, household well-being, economic security, and happiness.

More educated women are likely to score higher on the inner well-being dimensions since they are thought to be more in control over their lives. Also, it is likely that they have higher economic security because they might have other income beyond subsistence farming. They are likely to be less discriminated against, more respected, and to play a more active role in the community. Older women are likely to have more physical health problems but are expected to be more respected in the community.

Having a partner was regarded as a key element in a woman’s quality of life (Greco et al. 2015), hence the variable “married” is expected to have a positive association with all dimensions. Mothers who have a partner are likely to put less strain on their body (a lot of work is in agriculture) and hence to have better physical health compared to unmarried women. Though they are expected to be less in control over their lives, married mothers might feel less ashamed compared to single mothers, and hence the association with the inner well-being dimension is ambiguous. Despite women during the explorative research spoke about the brutality of intimate partner violence (and the DHS data reporting that nearly one of three married women had been a victim of violence in the past 12 months), the expected association between being married and family well-being is positive. Married women are more likely to enjoy better housing conditions, and her children are more likely to get an education. Married women are also more likely to be respected in the community, to have more economic security (strong positive association) and to be generally happier in their lives.

Women with higher wealth index scores^2^ are likely to have more bodily strength and to have less emotional worries. Moreover, they are expected to be better able to look after the other members of the household and to have a decent house. They are thought to be more respected in the community and to be happier and more satisfied with life.

### Discriminant Validity

Discriminant validity was tested using chi-squared tests on each dimension and on the overall score, aggregated using a normative approach (Greco 2016). Where the number of cell counts was less than 5, Fisher’s exact tests were used when computationally feasible; where they were not possible, values were grouped and variables were re-coded to increase cell counts.

It was hypothesized that quality of life is not related to the religion or ethnicity of the individual. There was no evidence from the qualitative study (Greco et al. 2015) or other sources (Colbourn, Masache, and Skordis-Worrall 2012) that having a particular religion or being part of a specific ethnic group affected overall quality of life, or any aspect of it.

### Convergent Validity

The WHOQOL-Bref has been chosen as the comparative measure for the WCI because it is a standard measure of quality of life, has been translated and validated in Chichewa, and used in Malawi to assess women’s quality of life (Colbourn, Masache, and Skordis-Worrall 2012). However, the WHOQOL-Bref is not grounded in the capabilities approach, and it has not been developed to measure capabilities; the selection of dimensions was not built with a bottom-up participative process (the first selection of domains was done by a panel of experts) (Group 1998). Thus, it makes it a good comparator for convergent validity: the two measures should show a good degree of correlations although not perfect.

The WHOQOL-Bref is composed of 26 questions grouped under 4 domains: physical domain, psychological domain, social relationship, and environment. For the purpose of this validity test, the scores of the four domains were aggregated giving equal weights to each domain. The aggregated score was calculated as the average of the four scores. The WHOQOL-Bref aggregated score was compared to the WCI, aggregated using four different methods (Greco 2016) for each individual. The correlations between the scores were explored using Pearson’s correlation coefficient. Convergent validity was tested on a subsample of 30 people, randomly selected from the main survey sample, representing approximately 10% of the total sample.

It is important to note that any measurement has some associated error; hence we should expect that correlations among indicators of the same attribute should be in the range of 0.4–0.8. Any lower correlation suggests that either the reliability of one or the other measure is likely to be unacceptably low, or that they are measuring different dimensions (Streiner and Norman 2008).

### Reliability

Reliability is the degree to which an instrument is free from measurement error. It was tested in two ways: internal consistency and test–retest.

#### Internal consistency

The test of internal consistency is the most widely used measure of reliability because it is the only one that can be derived with only one administration of the test. Consequently, many articles about instrument development report this test only and do not go further (Streiner and Norman 2008).

There is a need to derive some quantitative measure of the degree to which the items in the instrument are related to each other. Internal consistency of the instrument was tested for each item within each dimension and across dimensions. Cronbach’s alpha test (*α*) was used for testing the indicators within each dimension (consistency within dimensions). In addition, the correlation between each item and all the dimension scores (consistency across dimensions) was estimated using the Pearson product moment correlation coefficient.

Cronbach’s alpha tests the internal consistency by assessing the degree to which a set of items measure a single latent dimension (consistency within dimension). Alpha is equal to zero when the set of items measures different unrelated latent dimensions. When alpha is equal to or bigger than 0.70, it is considered acceptable (Nunnally, Bernstein, and Berge 1967; Baggaley et al. 2007; Nedjat et al. 2008; Webster 2010).

#### Test–retest reliability

Another step in providing evidence of the value of an instrument is to demonstrate that measurements of individuals at different times produce the same or similar results. The test–retest reliability was assessed following advice from the Guidelines for Evaluating and Expressing the Uncertainty of Measurement Results of the US National Institute of Standards and Technology (Taylor and Kuyatt 1994). A subsample of 30 respondents was randomly re-selected from the main survey, and interviewed a second time one month after the completion of the first interview. The last section of the questionnaire included an exercise which asked women to rank in order of preference from 1 (first) to 6 (last) the six capabilities, according to their own values (Greco 2016). The Pearson correlation coefficient was used to estimate the degree of correlation between the first and second rounds of ranking of the six capabilities.

## Results

### Content Validity

In general, the meaning of the questions was understood and interpreted by the respondents in the way the research team expected. A few cases of ambiguity or misinterpretation of the question were identified, where the wording had to be modified to reflect the true meaning. The quotes reported below present evidence in support of the instrument’s content validity.

Think-aloud interviews:

Interviewer: Do you believe in witchcraft?

Respondent: You want to know if I think that there is witchcraft in this village. I don’t believe in things that that I cannot see.

Interviewer: Do you ever feel ashamed of your appearance?

Respondent: This is how God made, so I am not ashamed.

Respondent: When I wear a poor “chitenge^3^” I do not want to go out and meet others, I feel ashamed.

Interviewer: Do you need to ask permission to go to the health clinic if you or your children are ill?

Respondent: When I am sick I do not have to wait for my husband, I can just go.

Paraphrasing:

Interviewer: In the past week, did you have time to relax and rest?

Respondent: You want to know for example if I was able to meet with friends for a chat.

Interviewer: Are you able to express your feeling freely?

Respondent: Am I able to put out what I have in my throat?

Interviewer: Do you feel oppressed?

Respondent: If you lack necessities, or you lack freedom of speech by your husband, then you are oppressed.

Respondent: You are oppressed if you are denied the chance to buy or trade, or if you are working without being paid.

### Construct Validity

Descriptive statistics for the sample are presented in Table 3, and the relationship between the socioeconomic characteristics and the dimensions of quality of life are presented in Table 4. Relationships that were anticipated are reported in italics. The direction of the relationship is noted in brackets, when negative. The remaining associations were tested for unexpected relationships and reported for completeness. Of the 20 anticipated non-ambiguous associations, 14 (70%) were confirmed to be statistically significant in the expected direction. The results revealed one association that was not hypothesized a priori.

**Table 3.** Socio-demographic characteristics of the household survey respondents (*n* = 258)

Variable	Category	Frequency	%
Area^a^	Rural	242	94.16
	Peri-urban	15	5.84
Age	<16	1	0.39
	16–20	20	7.75
	21–25	93	36.05
	26–35	87	33.72
	36–45	52	20.16
	46–55	5	1.94
Marital status	Married or with partner	219	84.88
	Single, divorced, or widow	39	15.12
Education	Never been to school	36	13.95
	Primary	193	74.81
	Secondary	29	11.24
Religion	CCAP^b^	23	8.95
	Roman Catholic	161	62.65
	Anglican	6	2.33
	Pentecostal or Adventist	48	18.68
	Other	19	7.39
Ethnic group	Chewa	230	89.15
	Ngoni	24	9.3
	Senga	3	1.16
	Other	1	0.39

^a^Measured as proximity to a trading centre or tarmac road.

^b^Church of Central Africa Presbyterians.

**Table 4.** Univariable association between dimensions of quality of life and socioeconomic characteristics

Socio economic variables	Physical strength	Inner well-being	Household well-being	Community relations	Economic security	Happiness	Quality of life
*Construct validity*							
Rural area	0.535	0.827	0.917	1.000	*0.109*	0.321	0.774
Health	n/a	0.002**	*0*.*316*	0.964	0.004**	0.005**	n/a
Education	0.093	0.000**	0.625	*0*.*457*	0.002**	0.193	0.001**
Age	*0.638*	0.638	0.830	*0*.*473*	0.740	(−) 0.034*	0.149
Married	0.001**	0.853	0.004**	0.001**	0.000**	0.016*	0.000**
Wealth index	0.002**	0.000**	0.000**	*0*.*140*	n/a	0.038*	n/a
*Discriminant validity*							
Religion	0.579	0.671	0.125	0.260	0.000**	0.403	0.005**
Ethnicity	0.633	0.883	0.174	0.052	0.305	0.766	0.894

Note: Cells in italic are those where an association was expected a priori*.*

*Significant (in the expected direction) at 5% level.

**Significant (in the expected direction) at 1% level.

The results suggest that there was no significant association between the Physical Strength dimension and the variable related to *age*; the Household Well-being dimension and the variable *health*; the dimension Community Relations and the variables *education*, *age,* and *wealth*. Moreover, association between the distance of the village to a main road and the economic stability of the woman appear to be not statistically significant.

Happiness was found to have unexpected relations with *age* (older women appeared to be less happy and less satisfied with their lives compared to younger women).

### Discriminant Validity

It was anticipated that the religious belief and the ethnic group of the respondents would have no association with any dimension of quality of life. As Table 4 reports, no significant relationship was found in the dimensions, except for one. The economic security component of the index was found to have an unexpectedly strong association with the respondent’s religion.

The relationship between the economic component of the index and these two individual characteristics was investigated further using the same correlation coefficient. The results in Table 5 show that people who belonged to the CCAP church scored significantly higher in the Economic Security component of the index, while Anglicans and other religions (such as Jehovah’s Witness) scored lower. However, these results should be interpreted with caution given the small sample size in each religious group category.

**Table 5.** Univariable association between religious faiths and the dimension Economic Security

Religion	Economic security
CCAP	0.008**
Roman Catholic	0.897
Anglican	(−) 0.004**
Pentecostal or Adventist	0.331
Other	(−) 0.015*

*Significant (in the expected direction) at 5% level.

**Significant (in the expected direction) at 1% level.

### Convergent Validity

The capability index aggregated with equal weights and the WHOQOL-Bref were compared. The correlation between the capabilities indices (aggregated using four different methods as described in (Greco 2016)) and the WHOQOL-Bref was explored using Pearson’s correlation coefficient (Table 6). The coefficients were considered acceptable because they were in the range 0.4–0.8.

**Table 6.** Correlation coefficients for the WHOQOL-Bref and the WCIs

	WHOQOL-Bref
Data-driven	0.6223
Equal	0.6104
Normative	0.6095
Hybrid	0.5669

### Reliability

#### Internal consistency

The highest correlation coefficient across the dimensions for each item is highlighted in bold in Table 7. All but one item were found to be mostly correlated to the dimension that they were assigned to, with the majority (85%) of the Pearson correlation coefficients within the acceptable range of 0.4–0.8. The variable related to food intake seemed to be more associated with the dimension Economic Security than Physical Strength.

**Table 7.** Correlation matrix of dimensions and sub-dimensions of the WCI

	Physical strength	Inner well-being	Household well-being	Community relations	Economic security	Happiness	Cronbach’s alpha
*Physical strength*							0.4518
Able body	**0.6555**	0.3513	0.1250	0.0243	0.1945	0.2228	
Food	0.4727	0.2233	0.3321	0.3908	**0.5088**	0.2770	
Avoid diseases	**0.5769**	0.2173	0.3230	0.3040	0.3692	0.2459	
Space births	**0.6316**	0.0261	0.1364	0.1396	0.1320	0.0831	
*Inner well-being*							0.6425
Inner peace	0.3166	**0.4277**	0.1328	0.1722	0.1197	0.1119	
Control	−0.0944	**0.3409**	0.0788	−0.0887	−0.1644	−0.0067	
Oppression	0.2894	**0.5687**	0.1860	0.1996	0.3237	0.3388	
Shame	0.2619	**0.6800**	0.2428	0.1825	0.3594	0.3104	
Knowledge	0.2097	**0.6509**	0.1381	0.0680	0.2905	0.1757	
*Household well-being*							0.587
Domestic violence	0.0791	0.1183	**0.3517**	0.2162	0.2213	0.1252	
Control money	−0.0719	0.0480	**0.3836**	0.0068	−0.0916	−0.1343	
House	0.2594	0.1941	**0.4932**	0.3736	0.4751	0.2811	
Edu. children	0.2058	0.2585	**0.5879**	0.1458	0.2787	0.2314	
Family care	0.3874	0.1221	**0.6750**	0.3207	0.4452	0.3439	
*Community relations*							0.6443
Access services	0.1416	0.0490	0.1212	**0.4833**	0.0971	−0.0267	
Safety	0.1635	0.0042	0.1380	**0.5190**	0.1083	0.0790	
Community group	0.1075	0.1785	0.1746	**0.3489**	0.1996	0.1112	
Discrimination	0.1435	0.0187	0.2323	**0.5035**	0.1446	0.0416	
Respect	0.2356	0.1768	0.3157	**0.7721**	0.4241	0.2486	
*Economic security*							0.7645
Safety net	0.3060	0.2836	0.2822	0.1901	**0.6434**	0.1937	
Land	0.1395	0.0904	0.0255	0.2054	**0.4025**	0.1911	
Asset	0.2082	0.3470	0.3167	0.2903	**0.6497**	0.3073	
Business	0.3231	0.1883	0.3511	0.2309	**0.6958**	0.3378	
Cope shock	0.3641	0.2513	0.4994	0.3725	**0.7106**	0.3922	
*Happiness*							0.8664
Satisfaction	0.2900	0.3241	0.2988	0.1617	0.4289	**0.9360**	
Happiness	0.3161	0.3077	0.3053	0.2152	0.4287	**0.9438**	
*Overall*							0.7385

Note: The highest correlation coefficient across the dimensions for each item is highlighted **in bold**.

Cronbach’s alpha values for each dimension ranged from 0.5 to 0.9. Happiness and Economic Security showed the greatest internal consistency with values of alpha greater than 0.7. Physical strength had the lowest internal consistency with the alpha just smaller than 0.5. The alpha coefficient on the overall index was at an acceptable level of 0.74.

#### Test–retest reliability

The ranking of the two rounds of the survey was compared for each of the 30 respondents using the Pearson correlation coefficient. The data showed an average level of reliability, with 63% of the retested rankings having a correlation coefficient above the acceptability threshold of 0.40.

## Discussion

This study investigated the validity and reliability properties of a newly developed measure for assessing women’s capabilities in Malawi. The measure was systematically tested for content, construct and convergent validity, internal consistency, and test–retest repeatability.

The content validity performed during the pilot process improved significantly the quality of the tool. The validity exercise was found to be very useful because, in addition to amending those questions that could be misunderstood or misinterpreted by respondents, it also clarified the meaning of the questions among the fieldworkers. Moreover, it emerged from field reports that respondents felt very much involved in the development of the tool, making it a truly “participatory” survey, in line with Sen’s ideals for social inclusion and democratic deliberation.

This extensive process of validation led to nearly all respondents answering all questions and almost 80% of people reported that none of the questions were difficult to answer. This is an indication of a high degree of acceptability and comprehensibility of the instrument. However, it was reported by the fieldworkers that the validation process was time-consuming and cognitively demanding for respondents as respondents are usually asked to limit their contribution to answering a question, and not to give feedback on the question itself.

The relationship between the socioeconomic characteristics and the dimensions of quality of life was investigated and compared with a priori expectations to investigate the construct validity of the measure. The majority of hypothesized associations (70%) were found to be statistically significant in the expected direction. This provides evidence that the instrument was measuring quality of life as intended in the conceptual model. Notable positive associations with the WCI were education and economic stability: more educated women appeared to have better business opportunities and to own more assets. Mothers living with a partner scored significantly higher in the family-related dimension, probably because they enjoyed better housing and the children had more chances to get educated and be well nourished. In contrast, data suggested that single mothers had more financial difficulties and were likely to be less able to cope with shocks.

Despite supportive findings for the majority of the constructs, a number of hypothesized relationships were not confirmed. It was expected that people living in remote villages would have fewer chances for business opportunities and would be more economically insecure; however, results suggested that there is no statistically significant association between the distance of the village to a tarmac road or trading centre and the woman’s economic stability. This might be due to the homogeneity of the sample: over 94% of respondents lived in rural areas; in fact, the geographical variable had no association with any dimension.

Although schooling, knowledge and material prosperity had been regarded by women in the FGDs as a valuable component in their lives, it appeared that more educated or wealthier women did not necessarily enjoy better community relations. The only significant driver of social status appeared to be having a partner, and not the assets owned or the number of years spent at school. These results are similar to a study that used the WHOQOL-Bref on the general population in a different part of the country (Colbourn, Masache, and Skordis-Worrall 2012).

Younger women were expected to have more bodily strength and to be less respected in the community compared to older women, but these associations were not found. A possible explanation could be that the age range of the sample was small (all women had had a baby in the previous year) and the majority of them (70%) were in the age range 21–35.

Spiritual beliefs and ethnic background were not expected to have an influence on the woman’s well-being. However, it is interesting to note that there was a highly significant relationship with religion: women who were part of the Church of Central Africa Presbyterian (CCAP) were more economically secure compared to those who were from other faiths. The CCAP was the first missionary church, established in Malawi with the arrival of the Scottish explorer David Livingstone in the second half of the nineteenth century. An explanation for this association could be that, despite not being the biggest religious group in Malawi (the DHS gives an estimate of membership of less than 17% of the population; National Statistical Office and ICF Macro 2011), it is the oldest and more settled, hence people who are part of it might be in a stronger financial position. Further anthropological research could provide better insights and understanding of these dynamics.

Additional evidence in support of the instrument’s validity was drawn from the distribution of the index, which appeared to be similarly distributed to an instrument measuring comparable (but not identical) domains of quality of life: the WHOQOL-Bref. Pearson’s coefficients between the two measures of quality of life showed a good degree of correlation implying that the two instruments were indeed measuring similar concepts.

The results from the internal consistency and test–retest repeatability offered reasonably encouraging evidence on the reliability of the instrument. All but one dimension had adequate internal consistency with both correlation coefficients and alpha scores at acceptable levels. All but one item were found to be mostly correlated to the dimension to which they were assigned, with the majority of the correlation coefficients greater than or equal to 0.40. This value is within the acceptability threshold given in other reliability studies (Baggaley et al. 2007; Nedjat et al. 2008; Webster 2010; Colbourn, Masache, and Skordis-Worrall 2012). The item which was not mostly correlated to the assigned dimension was the indicator related to food availability and consumption. This variable was mostly correlated with the dimension Economic Security. This might be due to the fact that people who were more likely to be food secure also had greater economic security and were more likely to be wealthier (Dreze and Sen 1989). This has implications for the reliability of the Physical Well-being dimension, which showed a lower degree of internal consistency compared to the other dimensions.

The test–retest exercise did not show a perfect correlation, suggesting that some people did change their responses when they were asked a second time to rank in order of importance the different aspects of their quality of life. This might be due to the challenge respondents faced when ordering dimensions that are all highly valuable. Respondents were asked at the end of the survey to indicate which question was the hardest to answer: the ranking exercise question was indeed found to be the most challenging. It could also be possible that respondents gave a different answer due to a change in their circumstances. Even so, the reliability coefficient is comparable to, or higher than, results generated in other studies in the health sector (Dong et al. 2003; Onwujekwe, Fox-Rushby, and Hanson 2005).

A limitation of this study is that the test–retest exercise was not done on the entire length of the questionnaire, but only on one section: the ranking exercise. It was felt that administering the whole questionnaire to the same respondent after a short period of time was too much of a burden for the woman, especially since she was asked to answer the WHOQOL-Bref during the same interview.

## Conclusions

This paper has provided the first rigorous and comprehensive validity testing of an innovative measure based on Sen’s capability framework, the WCI, which was developed with women in Malawi for assessing their quality of life. The results of the validity and reliability tests reported here provide supportive evidence that the WCI can be used as a robust evaluative tool for women’s quality of life in rural Malawi. Further research is currently under way to adapt the WCI for use in different contexts.

## Disclosure Statement

No potential conflict of interest was reported by the authors.

## Notes

1 International Society for Quality of Life Research.

2 The details of the asset index were presented in Greco et al. (2015).

3 “Chitenge” is a bright coloured cloth that women wrap around their waists as a long skirt.
